# Vitamin D Prevents Pancreatic Cancer-Induced Apoptosis Signaling of Inflammatory Cells

**DOI:** 10.3390/biom10071055

**Published:** 2020-07-15

**Authors:** Stefania Moz, Nicole Contran, Monica Facco, Valentina Trimarco, Mario Plebani, Daniela Basso

**Affiliations:** 1 Department of Medicine-DIMED, Laboratory Medicine, University of Padova, Via Giustiniani 2, 35128 Padova, Italy; nicole.contran@phd.unipd.it (N.C.); mario.plebani@unipd.it (M.P.); daniela.basso@unipd.it (D.B.); 2 Department of Medicine-DIMED, Hematology and Clinical Immunology Unit, University of Padova, 35128 Padua, Italy; monica.facco@unipd.it (M.F.); valentina.trimarco@unipd.it (V.T.)

**Keywords:** PDAC, immune system, vitamin D, intracellular calcium, cytokines, intracellular signaling pathways

## Abstract

Combined approaches based on immunotherapy and drugs supporting immune effector cell function might increase treatment options for pancreatic ductal adenocarcinoma (PDAC), vitamin D being a suitable drug candidate. In this study, we evaluated whether treatment with the vitamin D analogue, calcipotriol, counterbalances PDAC induced and SMAD4-associated intracellular calcium [Ca^2+^]_i_ alterations, cytokines release, immune effector function, and the intracellular signaling of peripheral blood mononuclear cells (PBMCs). Calcipotriol counteracted the [Ca^2+^]_i_ depletion of PBMCs induced by SMAD4-expressing PDAC cells, which conditioned media augmented the number of calcium flows while reducing whole [Ca^2+^]_i_. While calcipotriol inhibited spontaneous and PDAC-induced tumor necrosis factor alpha (TNF-α) release by PBMC and reduced intracellular transforming growth factor beta (TGF-β), it did not counteract the lymphocytes proliferation induced in allogenic co-culture by PDAC-conditioned PBMCs. Calcipotriol mainly antagonized PDAC-induced apoptosis and partially restored PDAC-inhibited NF-κB signaling pathway. In conclusion, alterations induced by PDAC cells in the [Ca^2+^]_i_ of immune cells can be partially reverted by calcipotriol treatment, which promotes inflammation and antagonizes PBMCs apoptosis. These effects, together with the dampening of intracellular TGF-β, might result in an overall anti-tumor effect, thus supporting the administration of vitamin D in PDAC patients.

## 1. Introduction

The known ability of the tumor microenvironment to influence tumor cell behavior appears to be of particular relevance in the fourth worldwide cause of cancer-related death, pancreatic ductal adenocarcinoma (PDAC), which is characterized by dense desmoplasia with dispersed immune cells, cancer associated fibroblasts (CAFs), and pancreatic stellate cells (PSCs). CAFs are the main effector cells in the desmoplastic reaction and PSCs, once activated by cytokines, growth factors, oxidative or metabolic stress, transdifferentiate to myofibroblast-like cells, and synthesize abundant extra cellular matrix (ECM) proteins [1,2]. Collagen fibers and stromal cells create a microenvironment that can counteract, but often favors, tumor growth and the selection of metastatic clones. Infiltrating immune cells comprise dendritic cells, CD4^+^ and CD8^+^ lymphocytes, regulatory T cells (T_regs_), tumor-associated macrophages (TAMs), and myeloid derived immunosuppressive cells (MDSCs). These cellular subsets are imbalanced towards immunosuppression, MDSCs and T_regs_ prevailing over dendritic cells and effector CD4^+^ and CD8^+^ lymphocytes. Since PDAC-associated immunosuppression concurs in reducing survival, the depletion of immunosuppressive cells and/or the restoration of immune effector cells might be effective strategies for improving outcome and be useful in treatments targeting molecules and pathways involved in immune cell regulation, such as those using checkpoint inhibitors with antibodies anti-CTLA4 or anti PD1/PDL1 [3]. However, as yet, these treatment strategies have proven disappointing in humans, and the factors underlying their failure are not well understood. Although the dense hypovascular stroma might antagonize drug and cell delivery by creating a physical barrier, within the two-way cross-talk between PDAC and stromal cells, the former by shaping immune cells might favor immunosuppression, thus compromising the therapeutic interventions designed to restore immune effector function. Novel combination approaches using drugs supporting immune effector cell function might be planned in order to overcome these limitations. A suitable candidate in this context appears to be vitamin D, which has been the object of intensive research in cancer, including PDAC, studies focusing on the role of vitamin D in cancer prevention on the one hand, and cancer treatment on the other [4].

Data from epidemiological studies report contradictory results, suggesting positive and negative associations between vitamin D and PDAC risk. In their pooled analysis of case-control studies, Waterhouse et al. found a 13% increase in PDAC risk for 100 IU/day vitamin D dietary intake, in agreement with Zablotska et al. who reported an increased PDAC risk in men whose dietary vitamin D intake was increased from 200 IU/day to 800 IU/day [5,6]. However, on analyzing two large prospective cohort studies, Skinner et al. found that vitamin D consumption of more than 600 IU/day lowers the risk of PDAC by 41% with respect to consumption of less than 150 IU/day [7]. In a large pooled analysis of data from 14 prospective cohort studies, Genkinger et al. concluded that there was not a positive nor a negative association between vitamin D intake and PDAC risk [8]. The search for an association between PDAC risk and plasma 25(OH)vitamin D levels has also yielded contradictory result: Wolpin et al. have reported an inverse, Stolzenberg-Solomon et al. in 2010 a positive, and Stolzenberg-Solomon et al. in 2009 no such correlation [9,10,11]. Despite these inconclusive data in the search for an association between vitamin D intake and/or vitamin D plasma levels and PDAC risk, the overall survival of PDAC patients with sufficient pre-diagnostic vitamin D plasma levels is longer than that of those with insufficient pre-diagnostic vitamin D plasma levels [12]. The beneficial effects of vitamin D supplementation on the survival of PDAC patients have been attributed not only to its direct anticancer effects [13], but also to its effects on the tumor microenvironment. In patients undergoing pre-operative chemoradiotherapy, Mukai et al. demonstrated that distant metastases-free survival was longer in those with higher than in those with lower levels of vitamin D, which, according to the authors, acts by suppressing the activation of CAFs [14]. In agreement, Sherman et al. demonstrated that vitamin D receptor engagement acts as a master transcriptional regulator of PSCs and, in association with standard gemcitabine treatment, prolongs survival in genetically engineered mice [15]. As well as CAFs and PSCs, myeloid cells might also be suitable targets of vitamin D. In a previous study, we demonstrated that PDAC derived exosomes induce MDSCs’ expansion by enhancing intracellular calcium trafficking, this effect being correlated with the loss of the tumor suppressor *SMAD4* gene [16].

The aim of the present study was to verify “in vitro” whether vitamin D might counteract PDAC induced *SMAD4*-associated intracellular calcium alterations, cytokines release, immune effector function, and the intracellular signaling of peripheral blood mononuclear cells (PBMCs), thus enhancing myeloid cell effector function.

## 2. Materials and Methods 

### 2.1. Cell Lines

The pancreatic cancer cell lines BxPC3 (American Type Culture Collection ATCC^®^ CRL-1687^™^, Rockville, MD, USA), known to carry a homozygous deletion of the *SMAD4/DPC4* gene, and BxPC3-*SMAD4+*, obtained from the BxPC3 cells stably transfected with the pBK-cytomegalovirus (CMV)-*SMAD4/DPC4* expression vector were used. The characterization of the cellular model, including the validation of transfection efficacy, has been described by us elsewhere [17]. The cell lines were cultured in RPMI 1640 (Thermo Fisher Scientific, Waltham, MA USA) supplemented with 10% fetal calf serum (FCS) (Thermo Fisher Scientific), 1% L-glutamine, and 0.1% gentamycin. One mg/mL Geneticin (G418 Sulphate) selective antibiotic (Thermo Fisher Scientific) was used only for the BxPC3-*SMAD4+* cell line. Three additional PDAC cell lines (Capan-1, PANC-1 and PSN-1) were used for flow cytometry analyses (Supplementary Materials and Methods).

### 2.2. Isolation of Human Peripheral Blood Mononuclear Cells

Human PBMCs were isolated from blood donors’ buffy coats by differential density gradient centrifugation (Histopaque^®^-1077, Sigma-Aldrich, Milano, Italy, F/H). After being washed twice with saline solution to remove contaminating platelets and centrifuged at 1200 rpm for 10 min, PBMCs were treated with a hemolysis solution (0.15 M NH_4_Cl, 10 mM KHCO_3_, 0.1 mM EDTA-Na_4_) for 10 min, centrifuged at 1200 rpm for 10 min, and finally used for the experiments.

### 2.3. Experimental Design

PBMCs were cultured in complete standard media (RPMI 1640, 10% FCS), in BxPC3 and BxPC3-*SMAD*4+ conditioned media (CM), in the presence or in the absence of calcipotriol (Calcipotriol hydrate, Sigma-Aldrich). After 2 culture days, PBMCs were collected for immunoblot analysis, and cytokines were measured in the supernatants after 2 and 4 days of culture. Intracellular calcium fluxes were analysed in the same experimental conditions after 2, 3, and 4 days of culture by epifluorescence (Fluo4-AM, Thermo Fisher Scientific) microscope analysis [16].

### 2.4. Immunoblot Analysis

Five million PBMCs were seeded in Petri dishes (ø 10cm) and cultured in complete standard media for 24h which was replaced with fresh complete standard media or BxPC3 and BxPC3-*SMAD*4+ CM in the presence or in the absence of 100 nM calcipotriol. Five million BxPC3 and BxPC3-*SMAD4+*were seeded in Petri dishes (ø 10cm) and cultured for 24h in complete standard media, which was replaced with fresh media added or not with 100 nM calcipotriol. After 2 days PBMCs and PDAC cell lines were collected for immunoblot analysis, which was performed as previously described by us [18], with the following primary antibodies diluted 1:3000in blocking buffer: anti-phospho-Akt (Ser^473^, Thr^308^), anti-Akt, anti-β-actin, anti-phospho-p44/42 MAPK (Erk1/2)(Thr^202^/Tyr^204^), anti-phospho-p38 (Thr^180^/Tyr^182^) anti-Cleaved Caspase-3 (Asp^175^), anti-Cleaved Caspase-8 (Asp^391^), anti-phospho-STAT-3 (Tyr^705^), anti-TGF-β, anti-phospho-NF-κB p65 (Ser^536^), anti-phospho-NF-κB p105 (Ser^933^)(Cell Signaling Technology, Danvers, MA, USA), anti-phosphoIκB-α (Ser^32^), and anti-IκB-α (Santa Cruz Biotechnology Inc., Santa Cruz, CA, USA). Secondary antibodies diluted 1:5000 in blocking buffer were: anti-rabbit (Cell Signaling Technology) or anti-goat (Sigma Aldrich). Each experiment was performed at least in triplicate.

### 2.5. Intracellular Calcium Flow Analysis

A total of 2 × 10^6^ PBMCs were seeded on coverslips that had been inserted in each well of six well culture plates and cultured for 2, 3, and 4 days in 2 mL of complete standard culture media or BxPC3 CM, BxPC3-*SMAD4+* CM in the presence or in the absence of 10, 100, and 1000 nM calcipotriol. Coverslips were then processed for the [Ca^2+^]_i_ fluxes study, as described by us elsewhere [19], using the intracellular calcium tracer Fluo-4 AMat 5 μM and an epifluorescence microscope. Four independent experiments, each made in triplicate, were performed. Intracellular fluorescence data obtained from any single cell, continuously monitored for 12 min (2.5 frames/sec), were analyzed considering the following: whole area under the curve, peak area, and the number of peaks using GraphPad Prism software, version 6.04 (San Diego, CA, USA). 

### 2.6. Cytokine Assay

TNF-α and TGF-β were measured in PBMCs supernatants after 2 and 4 days of culture in the above-described conditions by chemiluminescent immunometric assays (Immulite, Siemens Healthcare Diagnostic, UK) according to the manufacturer’s specifications. For all the experimental conditions, at least six independent sets of experiments were performed.

### 2.7. T-Lymphocyte Proliferation Assay

Lymphocytes were isolated from blood donors’ buffy coats by negative selection with RosetteSep™ Human T Cell Enrichment Cocktail (StemCell Technologies, Vancouver, BC, Canada), designed to isolate T cells from whole blood. Unwanted cells are targeted for removal with Tetrameric Antibody Complexes recognizing non-T cells and glycophorin A on red blood cells (RBCs). After a twenty-minute incubation of whole blood with the RosetteSep™, lymphocytes were isolated by gradient centrifugation (Histopaque^®^-1077, Sigma-Aldrich). For proliferation assay, T-lymphocytes were co-cultured with PBMCs (6 × 10^6^ cells per well in a 6-well plate) previously cultured in standard culture media, BxPC3 CM or BxPC3-*SMAD4+* CM in the presence or absence of 100 nM calcipotriol for 72 h. T-lymphocytes (50,000 cells) and PBMCs (50,000 cells) were resuspended in fresh standard culture media and seeded in a 96-well culture plate in the presence of 2.5 µg/mL phytohemagglutinin (PHA) (Sigma-Aldrich) and 100 U/mL of interleukin 2 (IL-2) (Chemicon, Prodotti Gianni, Milan, Italy), as proliferation stimulants for 72 h before [^3^H]-thymidine addition (1µMCi). After 10 hours ofco-culture, a scintillation counter (Model Tricarb 1600; Packard Instruments Company, Meriden, CT, USA) was used to measure [^3^H]-thymidine incorporation (counts per minute). A minimum of 12 replicate wells were counted for each experimental condition.

### 2.8. Flow Cytometry

Flow cytometry analyses for Annexin V expression was performed using BxPC3, BxPC3-*SMAD4+*, and three additional PDAC cell lines, as detailed in Supplementary Materials and Methods. 

### 2.9. Statistical Analysis

The statistical analysis of data was made using the Kruskal–Wallis rank test, Tukey’s multiple comparison test, analysis of variance (ANOVA), and Bonferroni’s adjustment of p value for multiple testing using Stata software, version 13.1 (StataCorp, Lakeway Drive, TX, USA).

## 3. Results

### 3.1. BxPC3, not BxPC3-SMAD4+ CM, Antagonizes Calcipotriol-Induced Intracellular Calcium Accumulation in PBMCs

Intracellular calcium flows of untreated and of 100 nM calcipotriol-treated PBMCs were studied after 2, 3, and 4 days of culture. For any time and any condition, single cell analysis of intracellular fluorescence was performed for 12 min. The number of peaks, the whole area under the curve defined by fluorescence data, and the peak area were calculated and statistically evaluated; the results are reported in Table 1. An increase in whole fluorescence areas indicates an overall intracellular calcium accumulation, while an increase in peak area indicates augmented intracellular calcium due to an increase in calcium flows. 

Whole and peak intracellular calcium areas were significantly reduced in untreated PBMCs after 3 days, with respect to 2 days of culture, while the number of intracellular calcium peaks significantly decreased after 4 days in untreated, and after 3 days in treated, PBMCs. Three days of culture were therefore chosen for the subsequent experiments, the aim being to ascertain the combined effects of pancreatic cancer and calcipotriol on the intracellular calcium pattern of PBMCs. Pancreatic cancer cell line-conditioned media caused different alterations in the intracellular calcium of PBMCs depending on the presence or complete absence of *SMAD4* gene: in the presence (not the absence) of *SMAD4*, the PDAC conditioned media induced an increase in the number of calcium peaks and a reduction in the whole intracellular calcium (Table 2). 

**Figure 1:**
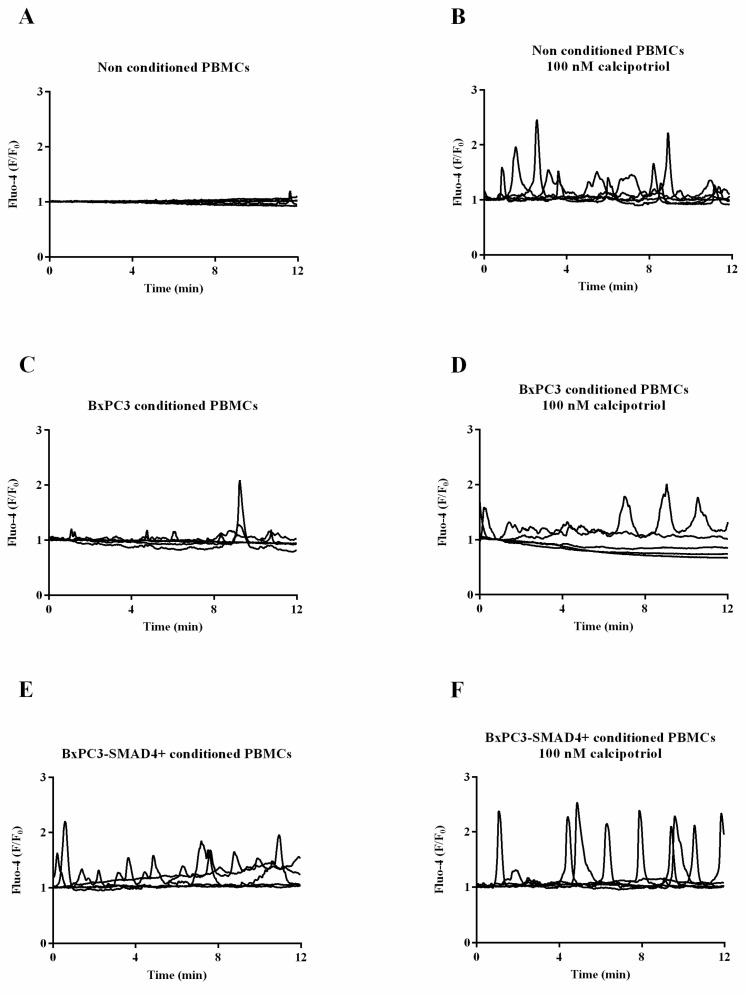
Intracellular calcium fluxes ([Ca^2+^]_i_) of PBMCs cultured or not with BxPC3 or BxPC3-*SMAD4+* conditioned media in the presence or in the absence of 100 nM calcipotriol.

The effects of increasing dosages of calcipotriol (10, 100, and 1000 nM) were then ascertained in non-conditioned and PDAC conditioned PBMCs (Table 3). In non-conditioned PBMCs, calcipotriol, mainly at the dosage of 100 nM, induced an increase in intracellular calcium, considering both the whole and the peak areas; representative experiments are shown in Figure 1 (panels A and B). Calcipotriol at 100 and 1000 nM had similar effects when PBMCs were cultured in BxPC3-*SMAD4+* conditioned media; representative experiments are shown in Figure 1 (panels E and F). Calcipotriol was not effective when PBMCs were cultured in BxPC3 conditioned media; representative experiments are shown in Figure 1 (panels C and D). 

### 3.2. Calcipotriol Reduces PBMC Release of TNF-α But Does Not Antagonize PDAC-Induced Lymphocytes Proliferation 

**Figure 2:**
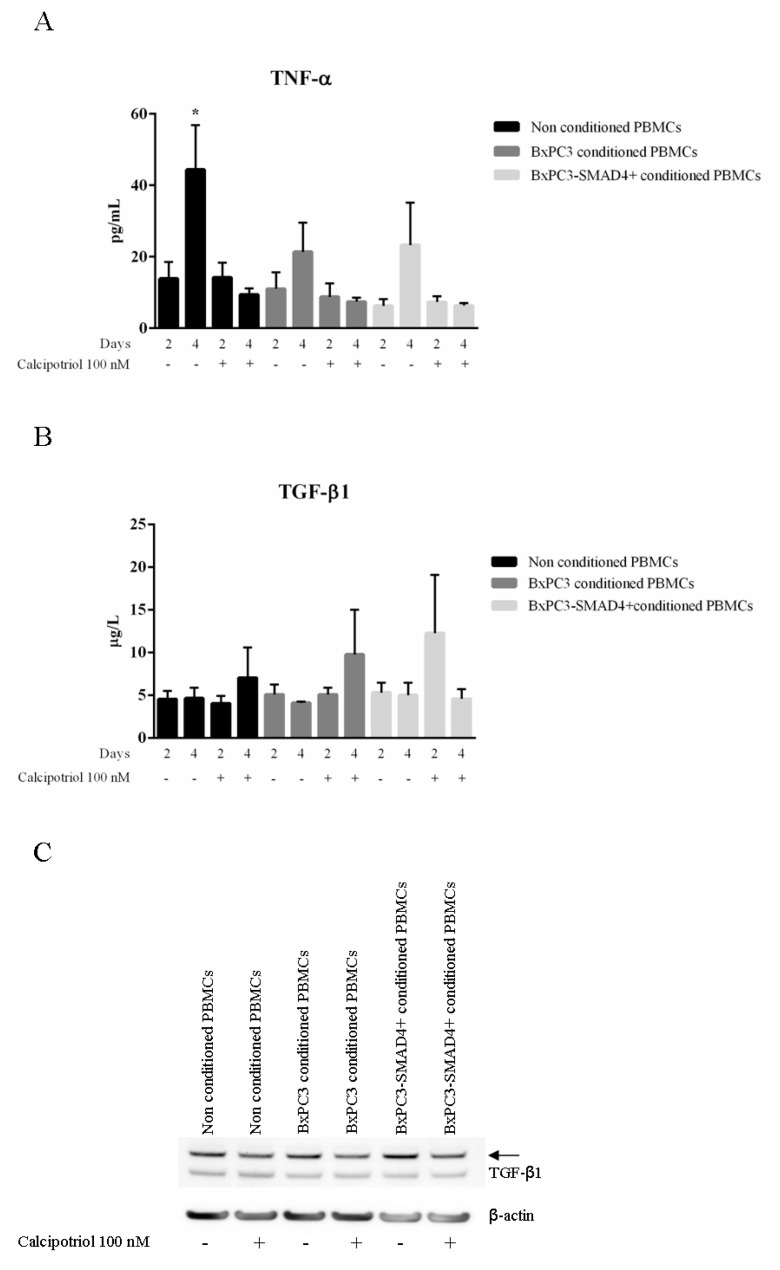
The levels of TNF-α and TGF-β were measured in PBMC supernatants cultured for 2 and 4 days in BxPC3 or BxPC3-*SMAD4+* conditioned media in the presence or in the absence of 100 nM calcipotriol (panels **A** and **B**). Western blot analyses show TGF-β protein level obtained from PBMCs cultured in the above-described conditions (panel **C**).

**Figure 3:**
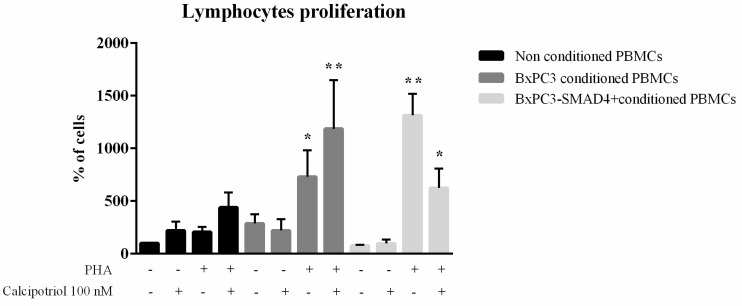
Lymphocytes’ proliferation. Columns show mean values, and bars, standard errors of lymphocyte proliferation after allogenic stimulation with PBMCs that remained non conditioned or were conditioned with BxPC3 or BxPC3-*SMAD4+* culture media. Non conditioned and conditioned PBMCs were not or were treated with 100 nMcalcipotriol. For all described conditions, lymphocytes’ proliferation was evaluated in the absence or in the presence of phytohemagglutinin (PHA) (2.5 µg/mL). Proliferation is expressed as a percentage with respect to values obtained from lymphocytes stimulated with allogenic nonconditioned and not calcipotriol treated PBMCs. Tukey’s multiple comparison test: * = *p* < 0.05 in comparison with BxPC3 conditioned PHA (-), calcipotriol (-) or (+) and with non conditioned PHA (+) calcipotriol (+); ** = *p* < 0.001 in comparison with BxPC3-SMAD4+ conditioned PHA (-) calcipotriol (-) or (+) and with nonconditioned PHA (+) calcipotriol (-).

In order to evaluate whether the observed variations in calcium pattern were correlated with the functional behavior of PBMCs, TNF-α and TGF-β were measured after 2 and 4 days in culture media of PBMCs treated as described above. TNF-α and TGF-β mean values with standard errors obtained from a minimum of three to a maximum of six independent experiments are shown in Figure 2 (panels A and B). In non-conditioned PBMCs, TNF-α significantly increased after 4 days in the absence, but not in the presence, of calcipotriol (Two-way analysis of variance: F = 5.197, *p* = 0.0157). When PBMCs were cultured in BxPC3 or BxPC3-*SMAD4+* conditioned media, TNF-α increased at 4 days in the absence of calcipotriol, although the variations were not statistically significant (F = 1.947, *p* = 0.1759 and F = 1.898, *p* = 0.1838, respectively). TGF-β tended to increase in the culture media of all PBMCs in the presence of calcipotriol, although the differences were not statistically significant (F = 0.4543, *p* = 0.7215 for non-conditioned PBMCs; F = 0.8877, *p* =0.4877 for BxPC3 conditioned PBMCs; F = 1.048, *p* = 0.4229 for BxPC3-*SMAD4+* conditioned PBMCs). TGF-β was further evaluated by Western blot analysis using PBMCs cultured for 2 days in the above-described conditions. Reduced TGF-β expression was observed when PBMCs, whether non-conditioned or PDAC conditioned, were cultured in the presence of calcipotriol (Figure 2, panel C). The effects of calcipotriol treatment of PBMCs on lymphocytes proliferation were then evaluated by co-culturing total lymphocytes in the presence of allogenic stimulation induced by non-conditioned or PDAC conditioned PBMCs, whether treated, or not, with calcipotriol. Lymphocytes proliferation was evaluated in the presence or absence of PHA. Figure 3 shows mean values with SEM of the obtained results. Co-culture of lymphocytes with PBMCs conditioned by PDAC media significantly enhanced PHA-stimulated lymphocyte proliferation, calcipotriol being found ineffective (F = 4.364, *p* = 0.0201 for conditioned media; F = 10.10, *p* < 0.0001 for calcipotriol treatment; F = 3.252, *p* = 0.0117 for interaction). 

### 3.3. Effects of PDAC Conditioned Media and Calcipotriol on Proliferation, Inflammation, and Apoptosis Signaling Pathways in PBMCs

**Figure 4:**
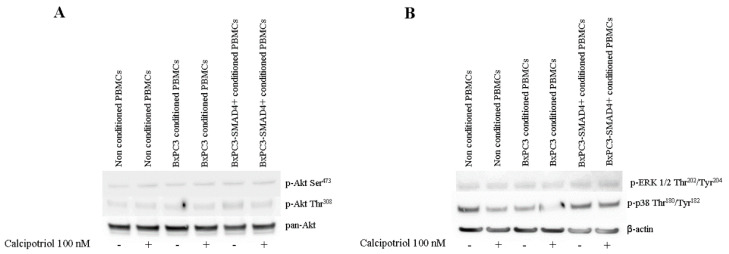
Western blot analyses obtained from PBMCs cultured or not cultured with BxPC3 or BxPC3-*SMAD4+*conditioned media in the presence or in the absence of 100 nM calcipotriol. Representative targets of the PI3K/AKT (panel **A**), ERK and p-38 (panel **B**) pathways are shown.

**Figure 5:**
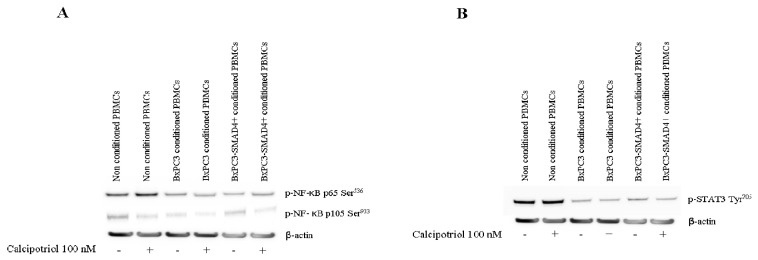
Western blot analyses obtained from PBMCs cultured or not cultured with BxPC3 or BxPC3-*SMAD4+* conditioned media in the presence or in the absence of 100 nM calcipotriol. Representative targets of the NF-κB (panel **A**) and STAT-3 (panel **B**) pathways are shown.

**Figure 6:**
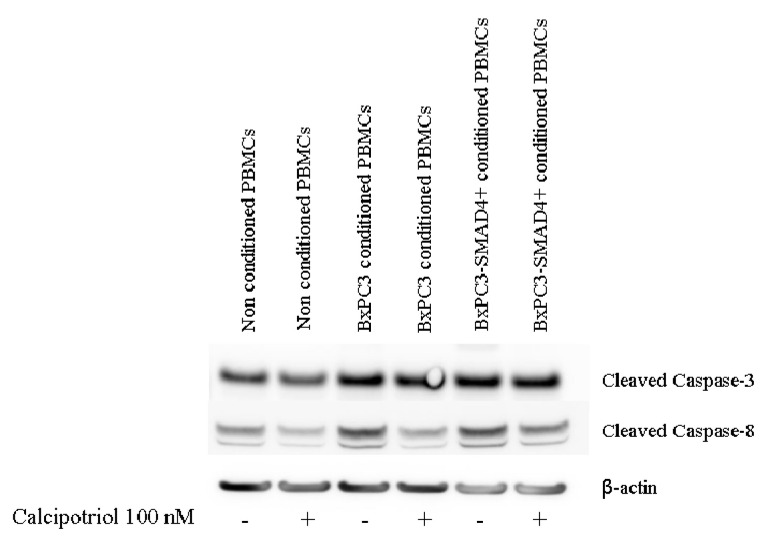
Western blot analyses obtained from PBMCs cultured or not cultured with BxPC3 or BxPC3-*SMAD4+* conditioned media in the presence or in the absence of 100 nM calcipotriol. Representative targets of Caspase pathway is shown.

**Figure 7:**
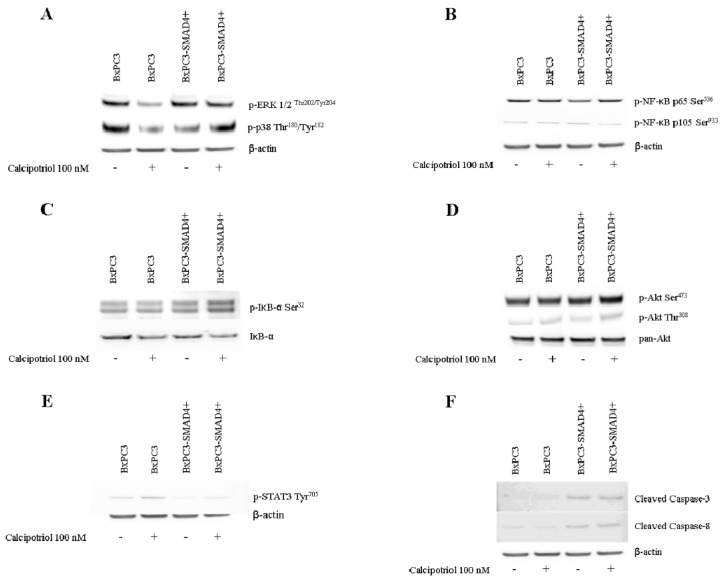
Western blot analyses obtained from BxPC3 or BxPC3-*SMAD4+* cell lines in the presence or in the absence of 100 nM calcipotriol. Representative targets of ERK and p-38 (panel **A**), NF-κB (panel **B**), IκB-α (panel **C**), PI3K/AKT (panel **D**), STAT-3 (panel **E**), and Caspase (panel **F**) pathways are shown.

The effects of PDAC conditioned media and of calcipotriol on PBMCs proliferation-, inflammation-, and apoptosis-related signaling pathways were then evaluated using Western blot analyses and flow cytometry. Figure 4 shows Western blot analyses of the proliferation-related targets p-AKT, p-ERK, and of p-p38 obtained from non-conditioned and from PDAC conditioned PBMCs, treated or not with calcipotriol for 2 days. Only p-p38 was influenced by PDAC conditioned media and by calcipotriol, BxPC3 CM and calcipotriol reducing p-38 phosphorylation. Figure 5 shows Western blot analyses of the inflammation-related targets NF-κB p-p65, NF-κB p-p105, and p-STAT-3 in the above-described conditions; p-IκB-α was undetectable in any condition (data not shown). Overall, PDAC conditioned media whether from cells expressing or not *SMAD4*, dampened NF-κB and STAT-3 pathways. Calcipotriol slightly activated NF-κB p-p65 in non-conditioned and in BxPC3-*SMAD4+* conditioned PBMCs. Figure 6 shows Western blot analyses of cleaved caspase 3 and cleaved caspase 8, involved in the apoptosis pathways, and Figure S1 shows the percentage of Annexin V. positive cells in the above-described conditions. Both caspases were activated by PDAC conditioned media, and this effect was counterbalanced by calcipotriol only when considering cleaved caspase 8. Almost half of PBMCs expressed Annexin V. Treatment with PDAC conditioned media and/or calcipotriol did not determine significant variations in the percentage of Annexin V expression. To ascertain the effects of calcipotriol on PDAC cells themselves, proliferation, inflammation, and apoptosis-related pathways were also evaluated by Western blot analyses and flow cytometry in BxPC3 and BxPC3-*SMAD4+* cells and in a further series of three PDAC cell lines, left untreated or treated with 100 nM calcipotriol for 2 days. Figure 7 and Figure S2 show the obtained results. Calcipotriol treatment determined a reduced phosphorylation of the proliferation related targets p-ERK and p-p38 in BxPC3 not in BxPC3-*SMAD4+* cells. Calcipotriol treatment determined a slight increase in the inflammation-related targets NF-κB (p-p65), p-IκB-α, and p-STAT-3 mainly in BxPC3-*SMAD4+* cells. Cleaved caspase 3 and 8 were undetectable in any studied condition. Annexin V was expressed by more than 50% of BxPC3 and PSN-1, in about 50% of Capan-1 and in less than 25% of BxPC3-*SMAD4+* and PANC-1. Calcipotriol treatment did not modify Annexin V expression by PDAC cell lines.

## 4. Discussion

The microenvironment is known to play a relevant role in the proliferation of pancreatic cancer and metastatic spread. Tumoral and stromal inflammatory cells interact and reciprocally modify their phenotype [20]. Tumor cells might shape the microenvironment, favoring a shift towards immunosuppression through the release of soluble or exosome-carried cytokines, chemokines and other mediators; in turn, stromal inflammatory cells might favor adjacent tumor cell growth, and prepare the niche for distant metastases. Elsewhere, we demonstrated that pancreatic cancer cells favor the expansion of MDSCs by targeting intracellular calcium [16]. Based on this observation, we suggested that vitamin D, a well-known intracellular calcium regulator, might support the maintenance of an effective anti-tumoral immune microenvironment by regulating intracellular calcium. 

We therefore verified whether calcipotriol, a vitamin D analogue, might balance the alterations in the intracellular calcium pattern induced by PDAC conditioned media on PBMCs, which were characterized by a reduced accumulation of intracellular calcium associated with an increase in the number of calcium flows when conditioned by PDAC cells expressing *SMAD4*, not by PDAC cells with the homozygous deletion (HD) for this tumor suppressor gene. Calcipotriol at both 100 and 1000 nM reverted this *SMAD4* associated effect, causing a significant intracellular calcium accumulation, similarly to that induced by calcipotriol in non-conditioned PBMCs. On the contrary, this vitamin D analogue had little, if any, effect on PBMCs conditioned by BxPC3 cells. These results indicate that *SMAD4* expression by PDAC cells determines not only a different effect on the intracellular calcium pattern of inflammatory cells, but also modulates the cellular response to calcipotriol treatment. Calcipotriol might thus evoke intracellular calcium accumulation in naïve PBMCs, in PBMCs previously shaped by PDAC cells expressing *SMAD4*, but not in PBMCs conditioned with PDAC cells HD for *SMAD4*. The loss of *SMAD4* by PDAC cells not only renders these cells more prone to proliferate and metastasize, but also confers on them the ability to render adjacent inflammatory cells less sensitive to intracellular calcium accumulation induced by calcipotriol. 

In order to ascertain whether these different responses in intracellular calcium might affect cellular function, we verified cytokine release by studying TNF-α and TGF-β. Other cytokines, namely IL-1β, IL-4, IL-6, and IL-10, were not evaluated since we had previously demonstrated that they are not detectable in this experimental setting [16]. As expected, with respect to non-conditioned PBMCs, the release of TNF-α was reduced by PDAC conditioned media. Interestingly, calcipotriol treatment abolished TNF-α release by both non-conditioned and conditioned PBMCs. Therefore, unlike the case of intracellular calcium, the inhibitory effect of calcipotriol on TNF-α release was not modified by PDAC. Since TNF-α is the main cytokine produced by dendritic cells, these findings suggest that calcipotriol might antagonize the differentiation of PBMCs into dendritic cells. This assumption was supported by our findings on the frequency of calcium peaks, which were reduced by calcipotriol treatment of PBMCs conditioned by *SMAD4*-expressing cells. Indeed, dendritic cells are characterized by the absence of calcium flows [16]. Moreover, BxPC3-*SMAD4+* conditioned and calcipotriol-treated PBMCs reduced PHA allogenic stimulation of lymphocyte proliferation, an effect not observed with BxPC3 conditioned media. PBMCs conditioned by these BxPC3 cells and treated with calcipotriol, unlike BxPC3-*SMAD4+*, presented no variations in the frequency of calcium flows and intracellular calcium accumulation, thus suggesting that this cell line has a negligible effect on dendritic cells. 

Of the different myeloid subsets comprised by PBMCs, MDSCs are extremely relevant in the biology of cancer as they exert their immunosuppressive effects by releasing immunosuppressive cytokines, the most relevant of which is TGF-β [21]. In our experimental settings, TGF-β tended to increase in culture media of non conditioned and conditioned PBMCs when treated with calcipotriol. This slight increase in soluble TGF-β was accompanied by reduced TGF-β intracellular levels. Overall, these findings indicate that activation of vitamin D pathway affects the TGF-β pathway, favoring TGF-β release from PBMCs intracellular storesthus favouring tumor protection in the early stages [22,23,24]. 

Calcipotriol effects on intracellular signaling pathways involved in proliferation, inflammation, and apoptosis were also investigated at 100 nM concentration, which affects intracellular calcium and is comprised within the serum reference interval [25]. The constitutive activation of the pro-proliferative ERK signaling pathway, observed in both BxPC3 and BxPC3-*SMAD4+*cells, was antagonized in part by calcipotriol, mainly in BxPC3 cells. In PBMCs, this pathway and the Akt pathway were not constitutively activated nor were they activated by PDAC conditioned media or by calcipotriol. These results reflect the absence of PBMCs proliferation [16] and suggest that it is not inducible by PDAC.BxPC3 and BxPC3-*SMAD4+* cells also showed the constitutive activation of the NF-κB pathway. Calcipotriol slightly induced IκB-α phosphorylation in BxPC3-*SMAD4+* cells and this finding suggests a cautious evaluation in Vitamin D administration since NF-κB is known to play a pivotal role in pancreatic cancer proliferation, metastases, and chemoresistance [26]. This cautious approach should be followed also because calcipotriol was unable to counteract the PDAC-caused inhibition of the inflammatory pathways NF-κB and STAT-3 in PBMCs. Further in vivo studies to confirm the effect of Vitamin D and calcium administration on PDAC cells are therefore advised.

Cell proliferation is in homeostasis with apoptosis in normal tissues, but they are imbalanced in cancer. We evaluated the intrinsic and extrinsic apoptosis-related pathways by the Western blot analyses of cleaved caspase 3 and cleaved caspase 8 [27].Very faint signals were found in PDAC cells, which conditioned media were otherwise able to induce caspase 3 and caspase 8 cleavage in PBMCs [28]. These data suggest that PDAC cells are resistant to apoptosis, but they can activate apoptosis signals in adjacent stromal cells. Within this scenario, calcipotriol appeared ineffective in inducing PDAC cells apoptosis, as verified also by Annexin V expression. Calcipotriol only limited caspase 8 cleavage in PDAC conditioned PBMCs, but it did not modify Annexin V expression. It should be noticed that although caspases were not activated, a high percentage of PDAC cells expressed Annexin V. This discrepancy is in line with previous observations made with ovarian carcinoma cells in peritoneal and pleural effusions [29], and is supported also by previous data indicating that externalization of Annexin V might occur independently from apoptosis, a sustained elevation of cytosolic calcium being one of the mechanisms involved [30].Another correlated cell process that should be evaluated in further studies is autophagy, a mechanism that allows pancreatic cancer cells to survive within adverse environmental conditions [31]. 

Calcipotriol therefore acts more on inflammatory cells than on PDAC cells. In PBMCs, calcipotriol dampens TNF-α, induces TGF-β in low amounts, and counterbalances PDAC-induced apoptosis. Overall, these data support calcipotriol as a drug of potential benefit in PDAC treatment, through its actions on cytokines and immune cells.

## 5. Conclusions

In conclusion, alterations induced by PDAC cells in the intracellular calcium of immune cells can be partially reverted by the administration of calcipotriol, which tends to restore PDAC-inhibited NF-κB signaling and antagonizes apoptosis. These effects, together with the induced TGF-β release in very low amounts, might result in an overall anti-tumoral response, thus supporting the clinical use of vitamin D in PDAC patients, even if pancreatic cancer cells appear insensitive to vitamin D treatment. 

## Figures and Tables

**Table 1:** Effects of time and calcipotriol on the intracellular calcium [Ca^2+^]_i_ of PBMCs. Untreated and 100 nM calcipotriol-treated PBMCs were studied after 2, 3, and 4days of culture. Data are from four independent experiments, and the total number of analyzed single cells is reported in brackets.

		Untreated PBMCs			Calcipotriol Treated PBMCs		
Time		2 days (*n* = *32*)	3 days (*n* = *58*)	4 days (*n* = *32*)	2 days (*n = 52*)	3 days (*n = 68*)	4 days (*n = 30*)
**Number of [Ca^2+^]_i_ Peaks**	Median	2	1	1 ^#^	3	1 ^*^	1 ^*^
	10^th^–90^th^ percentiles	1–4	1–4	1–1	1–12	1–4	1–2
	Kruskal–Wallis test	*p* = 0.0001			*p* = 0.0001		
**Whole [Ca^2+^]_i_ Area**	Median	31	16 ^§^	34	14	16	13
	10^th^–90^th^ percentiles	17–59	6–30	21–52	5–57	7–83	8–23
	Kruskal–Wallis test	*p* = 0.0001			*p* = 0.0966		
**Peak [Ca^2+^]_i_ Area**	Median	17	11 ^*^	13	8	8	5
	10^th^–90^th^ percentiles	5–41	3–20	7–17	2–25	1–54	3–10
	Kruskal–Wallis test	*p* = 0.0099			*p* = 0.1284		

Wilcoxon p values adjusted for multiple comparisons: ^#^ = *p* <0.05 with respect to 2 and 3 days; ^*^ = *p* <0.05 with respect to 2 days; ^§^ = *p* <0.05 with respect to 2 and 4 days.

**Table 2:** The effects of pancreatic ductal adenocarcinoma (PDAC)conditioned media on the intracellular calcium [Ca^2+^]_i_ of PBMCs. PBMCs were cultured for 3 days in non-conditioned medium, BxPC3, or BxPC3-*SMAD4+*conditioned media. The data reported are from four independent experiments, and the total number of single cells analyzed is reported in brackets.

.		Non Conditioned PBMCs (*n = 94*)	BxPC3 Conditioned PBMCs (*n = 52*)	BxPC3-*SMAD4+*Conditioned PBMCs (*n = 17*)
**Number of [Ca^2+^]_i_ Peaks**	Median	1	1	5 *
	10^th^-90^th^ percentiles	1–4	1–3	1–7
	Kruskal–Wallis test	*p* = 0.0001		
**Whole [Ca^2+^]_i_ Area**	Median	17	21	8 *
	10^th^-90^th^ percentiles	5–45	10–80	5–20
	Kruskal–Wallis test	*p* = 0.0001		
**Peak [Ca^2+^]_i_ Area**	Median	8	11	7
	10^th^-90^th^ percentiles	1–27	3–34	3–11
	Kruskal–Wallis test	*p* = 0.0348		

Wilcoxon p values adjusted for multiple comparisons: * = *p* <0.05 with respect to non-conditioned and BxPC3 conditioned.

**Table 3:** The effects of PDAC and calcipotriol on the intracellular calcium [Ca^2+^]_i_ of PBMCs. PBMCs were cultured in non-conditioned medium, BxPC3 or BxPC3-*SMAD4+* conditioned media for 3 days in the absence (0 nM) or presence of increasing dosages of calcipotriol (10, 100, 1000 nM). Data are from four independent experiments, the total number of single cells analyzed being reported in brackets.

		Non Conditioned PBMCs				BxPC3 Conditioned PBMCs				BxPC3-*SMAD4+*Conditioned PBMCs			
Calcipotriol Dosage		0 nM (*n = 94*)	10 nM (*n = 26*)	100 nM (*n = 61*)	1000 nM (*n = 28*)	0 nM (*n = 52*)	10 nM (*n = 55*)	100 nM (*n = 60*)	1000 nM (*n = 17*)	0 nM (*n = 17*)	10 nM (*n = 42*)	100 nM (*n = 27*)	1000 nM (*n = 31*)
**Number of [Ca^2+^]_i_ Peaks**	Median	1	1	1	1	1	1	1	1	5	1 ^#^	3	2
	10^th^-90^th^ percentiles	1–4	1–3	1–4	1–3	1–3	1–4	1–4	1–4	1–7	1–4	1–6	1–5
	Kruskal-Wallis test	*p* = 0.2814				*p* = 0.9430				*p* = 0.0001			
**Whole [Ca^2+^]_i_ Area**	Median	17	26	50 **	22	21	21	30	20	8	14	48 ***	27 ***
	10^th^-90^th^ percentiles	5–45	11–102	11–110	11–78	10–80	10–63	11–174	11–156	5–20	6–33	12–171	12–96
	Kruskal-Wallis test	*p* = 0.0001				*p* = 0.0567				*p* = 0.0001			
**Peak [Ca^2+^]_i_ Area**	Median	8	9	22 *	14	11	9	16	16	7	7	29 ***	23 ***
	10^th^-90^th^percentiles	1–27	1–94	5–64	1–42	3–34	2–20	2–82	4–92	3–11	3–24	8–77	7–77
	Kruskal-Wallis test	*p* = 0.0001				*p* = 0.0099				*p* = 0.0001			

^#^ = *p* <0.05 with respect to calcipotriol 0nM and 100 nM. * = *p* <0.05 with respect to calcipotriol 0 nM. ** = *p* <0.05 with respect to calcipotriol 0 nMand 1000 nM.*** = *p* <0.05 with respect to calcipotriol 0 nMand 10 nM.

## Supplementary Materials

Flow cytometry analysis. The following are available online at https://www.mdpi.com/2218-273X/10/7/1055/s1, Figure S1: Flow cytometry analysis of PDAC cells, Figure S2: Flow cytometry analysis of PBMCs.
