# Supplementary material for: Vitamin D Prevents Pancreatic Cancer-Induced Apoptosis Signaling of Inflammatory Cells
Source: Biomolecules. 2020 Jul 15;10(7):1055. doi: 10.3390/biom10071055 (PMC7408286; doi:10.3390/biom10071055)
Supplement: Supplementary file 1 [file biomolecules-10-01055-s001.pdf]

Supplementary Materials

Cell lines

The pancreatic cancer cell lines used for Flow cytometry analysis were: BxPC3, BxPC3-SMAD4+, Capan-1 (American Type Culture Collection ATCC® HTB-79™ Rockville, MD, USA), PANC-1 (ATCC® CRL-1469™) and PSN-1 (ATCC® CRM-CRL-3211™). All cell lines were cultured in RPMI 1640 (Thermo Fisher Scientific, Waltham, MA USA) supplemented with 10% (BxPC3, PANC-1 and PSN-1) and 20% (Capan-1) fetal calf serum (FCS) (Thermo Fisher Scientific), 1% L-glutamine and 0.1% gentamycin. One mg/mL Geneticin (G418 Sulphate) selective antibiotic (Thermo Fisher Scientific) was used only for the BxPC3-SMAD4+ cell line.

Flow cytometry analysis

5 × 105 of PBMCs were seeded in 6 well/plate and cultured in complete standard media for 24 hours which was replaced with fresh complete standard media (NC) or BxPC3, BxPC3-SMAD4+, Capan-1, PANC-1 and PSN-1 CM in the presence or in the absence of 100 nM calcipotriol. After 2 days the cells were collected, leaving intact the adherent layer, and examined for apoptosis status by staining with Annexin V-FITC and Propidium Iodide (PI) accordingly to the manufacturer's instructions (Immunostep; Salamanca, Spain). The cells were harvested, washed and 100 μL of binding buffer plus 5 μL of Annexin V-FITC were added for further 10 minutes in the dark and at RT. After the incubation, 100 μL of binding buffer were added and cells were analyzed by flow cytometer FACS CantoII. At least 20,000 events were collected using FACS Diva software. One million of BxPC3, BxPC3-SMAD4+, Capan-1, PANC-1 and PSN-1 were seeded in Petri dishes (ø 10 cm) and cultured for 24 hours in complete standard media, which was replaced with fresh media added or not with 100 nM calcipotriol. After 2 days the cells were collected and apoptosis analysis was performed in the same conditions described above.

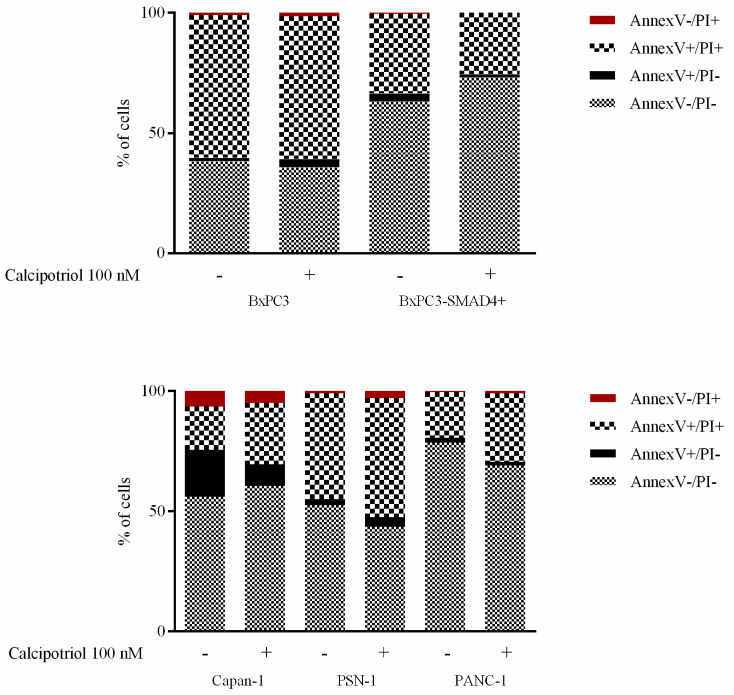

Figure S1. Flow cytometry analysis of PDAC cells cultured in the presence or in the absence of 100 nM calcipotriol. The figure shows the percentage of live (Annexin V-/PI-), early apoptotic (Annexin V+/PI-), late apoptotic (Annexin V+/PI+) and necrotic (Annexin V-/PI+) cells.

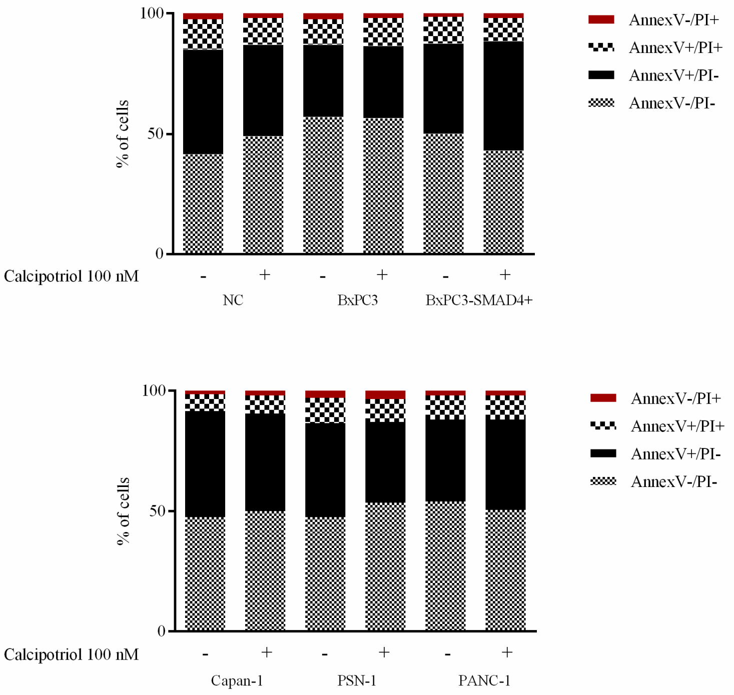

Figure S2. Flow cytometry analysis of PBMCs cultured in non conditioned (NC) or in PDAC conditioned media in the presence or in the absence of 100 nM calcipotriol. The figure shows the percentage of live (Annexin V-/PI-), early apoptotic (Annexin V+/PI-), late apoptotic (Annexin V+/PI+) and necrotic (Annexin V-/PI+) cells.
